# Practical Method for Evaluating the Element Sensitivity Variation of an Ultrasonic Annular Phased Array Transducer

**DOI:** 10.3390/s26010025

**Published:** 2025-12-19

**Authors:** Zhengxiao Sha, Xiao Liu, Yanze Liu, Xiao Wang, Xiaoming Zhou

**Affiliations:** 1 AECC Beijing Institute of Aeronautical Materials, Beijing 100095, China; liuxiao621@126.com (X.L.);; 2 Beijing Key Laboratory of Aeronautical Materials Testing and Evaluation, Beijing 100095, China; 3 Key Laboratory of Science and Technology on Aeronautical Materials Testing and Evaluation, Aero Engine Corporation of China, Beijing 100095, China

**Keywords:** annular phased array transducer, Rayleigh integral, sensitivity variation

## Abstract

The unique features of annular phased array transducers, such as ring-shaped elements and the concentric configuration, cause them to behave differently from commonly used linear array transducers, in terms of sound field distribution and pulse–echo response. Consequently, standard techniques for assessing linear array transducers can introduce significant errors when applied to annular array transducers, especially concerning element-to-element sensitivity variance. This study investigates the consistency of element sensitivity in annular phased array transducers. Through theoretical analysis, a Long-Belt source assumption model was developed based on the Rayleigh integral to characterize the responses of ring-shaped elements in an analytical and explicit form. The model suggests that the response amplitude is linearly correlated with the radial width of the element, which was validated by subsequent numerical simulations. Based on these findings, a modified sensitivity evaluation algorithm for annular array transducers is presented. The response voltage per unit width, rather than the total response voltage, is used to eliminate the influence of varying geometries and sizes across elements. The sensitivity variation of a 32-element annular array transducer was evaluated using the new algorithm. Compared to the uncorrected measurement, the maximum sensitivity variation was reduced significantly from 25 dB to 6 dB, revealing the transducer’s intrinsic consistency despite the different geometric features of each element. Due to its distinct geometry compared to the ring-shaped elements, the central element cannot be corrected or evaluated using this method. These results suggest that the proposed algorithm enables the more accurate evaluation of sensitivity consistency for annular phased array transducers, thereby improving measurement reliability in practical applications.

## 1. Introduction

Phased array ultrasonic testing (PAUT) is an advanced non-destructive testing (NDT) method [1] based on the coordinated action of multiple array elements. It achieves the dynamic deflection, focusing, and sweeping of the sound beam by controlling the excitation timing and amplitude of each element. Due to its high resolution, flexibility, and efficiency, PAUT is widely used [2,3,4,5,6] in aerospace, petrochemical, energy, and other industrial fields. The multi-element nature of PAUT enables the acquisition and processing of massive amounts of data simultaneously. Integrating with technologies such as machine learning [7,8] and full matrix capture (FMC) [9] enables more efficient and intelligent non-destructive testing.

Compared with linear and rectangular arrays, PAUT based on annular arrays has received less attention. Due to the concentric arrangement of its elements, the annular phased array enables spherical focusing of the sound beam, as opposed to the cylindrical focusing characteristic of linear arrays. This overcomes the limitation of traditional linear arrays, which can only focus cylindrically, thereby intensifying the energy concentration. Therefore, a better signal-to-noise ratio (SNR) can be achieved in the focal zone due to a stronger focusing effect. Due to its unique sound field manipulation capabilities and high sensitivity, the annular phased array offers significant technical advantages in certain applications [10]. For example, annular phased arrays have been used in the ultrasonic testing of thick-section metal components, such as engine turbine disks [11,12,13]. With dynamic focusing and a large aperture, this technique achieves high-sensitivity and high-efficiency detection at full depths of over 100 mm. Additionally, the annular phased array technique has been applied to detect internal defects in tubes, shaft components, and composite materials. In recent studies [14,15], the annular array has been used in combination with full matrix capture (FMC) and the total focusing method (TFM), achieving promising results.

As the industrial application of PAUT continues to expand, the requirements for testing reliability and consistency have correspondingly increased. Among all factors, the ultrasonic transducer [16,17,18] has a non-negligible influence on the testing results, especially the sensitivity consistency of the phased array elements, which directly affects the integrity and energy distribution of the synthesized sound beam. The non-uniformity in element performance can generate or amplify [19] beam artefacts such as side lobes and poor resolution, and result in potential failure of detecting hazardous defects. In the field of industrial NDT, the evaluation of the element sensitivity consistency is vitally important to ensure an array transducer is fit for purpose.

Numerous specifications and standards are available to evaluate the performance of PAUT probes, notably ISO 18563-2 [20] and ASTM E2904 [21]. Both standards specify the characterization tests, methodologies, and acceptance criteria for array probes, and, notably, they mandate periodic evaluation of sensitivity consistency across all elements. For example, as a mandatory clause of ASTM E2904-22, the sensitivity variation of each element shall be measured and evaluated, and the results shall fall within specified limits.

**Figure 1:**
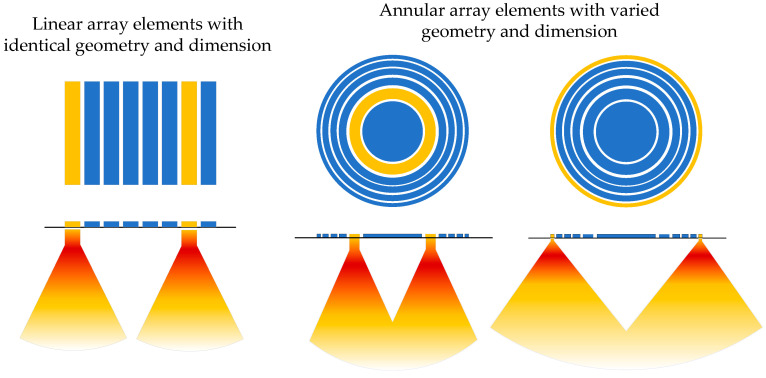
Illustration of the different structural features and their radiated sound fields of linear array and annular array transducers (Yellow elements are activated).

While the calibration method for linear array transducers [22,23] was extensively investigated, less attention was received in the annular array transducers performance evaluation. Because each element in a linear array transducer has an identical shape and size, sensitivity consistency can be evaluated straightforwardly by measuring and comparing the echo amplitudes from a planar reflector for each element. Unfortunately, this approach is not applicable to annular phased array transducers. These transducers consist of a series of concentric ring elements, each with unique inner and outer diameters. Due to these structural differences, the sound field distributions can vary drastically [15,23,24,25,26] because of the unique geometry of each element, as shown in Figure 1. For instance, the central circular element acts as a typical piston source, while the outer rings tend to generate a more diverged sound field.

Therefore, the sensitivity depends not only on the element’s electro-acoustic conversion efficiency but also on its specific sound field distribution. Consequently, an annular array transducer, even if manufactured with perfectly uniform electro-acoustic characteristics, will fail to meet the sensitivity variation criteria when calibrated with the same methodology as a linear array transducer. Manufacturers and users are faced with a dilemma: either lower the acceptance criteria and accept probes of poor quality, or modify the probe design to reduce the sensitivity differences caused by geometric structures.

This paper introduces a simplified model, based on theoretical analysis, to describe the behavior of ring-shaped transducers operating in pitch–catch mode. We performed simulations using both the proposed model and the Rayleigh integral to investigate the sound field distribution and response characteristics. Based on ISO 18563-2, the performance characterization standard for phased array transducers, we propose a corrected algorithm to eliminate the geometry induced sensitivity variation of an annular array transducer. Finally, experiments were conducted using a 32-element annular array transducer to validate the effectiveness and feasibility of the proposed algorithm.

## 2. Theoretical Analysis

### 2.1. Response of a Ring-Shaped Transducer

**Figure 2:**
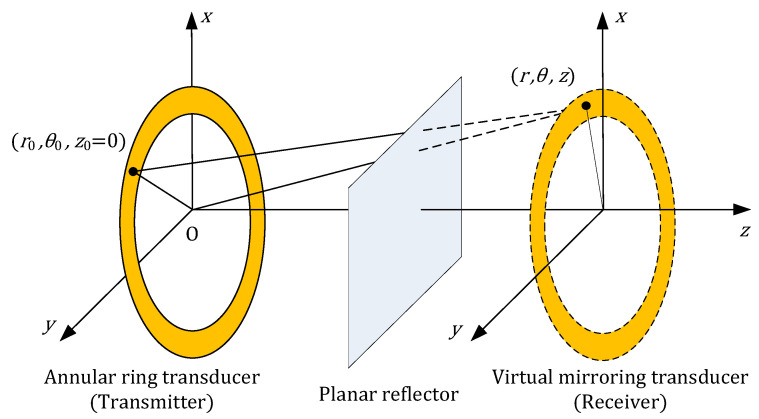
Measuring the response of an annular ring-shaped transducer from a planar reflector.

For a single annular transducer, the sensitivity measurement scenario can be described as follows. The transducer, located in the plane *z* = 0, transmits ultrasound into the *z* > 0 half-space. The ultrasound is then reflected by an infinite planar reflector at *z* = *d* and received by the same transducer. To simplify the analysis, we neglect reflection loss and model the scenario in pitch–catch mode rather than pulse–echo mode. A virtual mirror transducer, identical to the transmitter, is placed at *z* = 2 *d* to act as the receiver, as shown in Figure 2.

For an arbitrary acoustic source, the displacement potential at any point *P* can be calculated by means of Rayleigh integral,

(1) $$p\left(x,y,z\right)=\frac{-i\omega \rho }{2\pi }\iint_{S}v_{n}\frac{e^{ikr}}{r}dS$$

where *p* is the displacement potential of the point of interest *P*, $\left(x,y,z\right)$ are the cartesian coordinate, ω is the angular frequency, ρ is the density of the medium, $v_{n}$ is the normal velocity of integral unit area *dS*, k=ω/c is the wave number, *c* is the wave speed in the medium, $r=\sqrt{{\left(x-x^{\prime }\right)}^{2}+{\left(y-y^{\prime }\right)}^{2}+z^{2}}$ is the distance from *P* to *dS*.

As far as an annular ring transducer is concerned, the equation can be rewritten in the cylindrical coordinates as

(2) $$p\left(r,z\right)=\frac{-i\omega \rho v_{0}}{2\pi }\int_{a}^{b}\int_{0}^{2\pi }\frac{e^{ik\sqrt{r^{2}+r_{0}^{2}-2r_{0}rcos\theta_{0}+z^{2}}}}{\sqrt{r^{2}+r_{0}^{2}-2r_{0}rcos\theta_{0}+z^{2}}}r_{0}d\theta_{0}dr_{0}$$

where $v_{0}$ is the velocity of the transducer, *a* and *b* are the inner and outer radii of the ring, respectively, *r* and *z* are the radial and axial position of an arbitrary point in space, $\left(r_{0},\theta_{0},z_{0}=0\right)$ is the cylindrical coordinates of the integral point on the transducer.

As long as the spatial sound field distribution of Equation (2) is obtained, the pulse–echo signal could be calculated through another Rayleigh integral,

(3) $$V(\omega )=S(\omega )\int_{a}^{b}\int_{0}^{2\pi }p\left(r,z\right)rd\theta dr$$

where V(ω) is the voltage response of frequency ω, S(ω) is the coefficient related to the acoustic–electric transition.

### 2.2. Long-Belt Source Assumption

Unfortunately, no analytical solution exists for the integrals in Equations (2) and (3). Although well-established models such as the Spatial Impulse Response (SIR), the Pencil model, and the Finite Element Method (FEM) are available to address this problem [27,28,29,30,31,32], they require extensive numerical calculations to achieve satisfactory accuracy. This is particularly true for annular arrays, which consist of tens of elements with varying geometries and sizes. Therefore, it is preferable to find a simpler, more feasible method for revealing this relationship analytically and explicitly.

**Figure 3:**
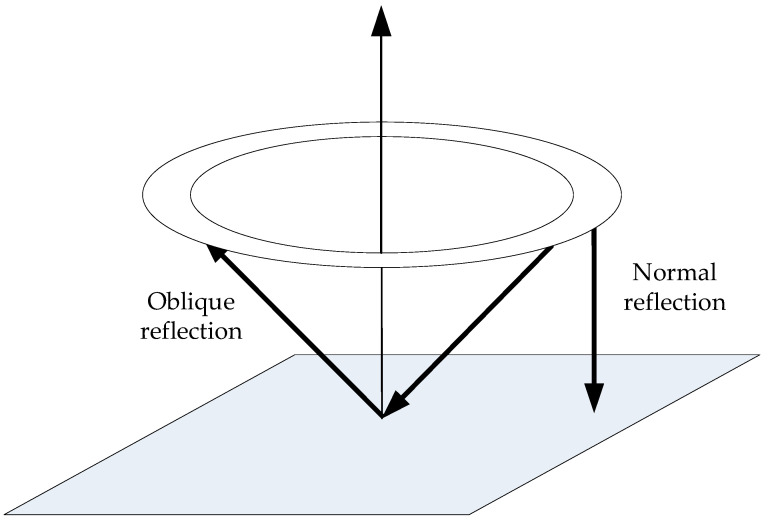
Illustration of the normal reflection and the oblique reflection.

In some cases, the far-field or the near-axial approximations [33,34] may be used to simplify the calculation. However, neither method is suitable for the situation discussed herein, as the distance between the transducer and the reflector must be kept sufficiently small. First, the echo signal from a single annular element is weak and is significantly attenuated over a long acoustic path in water. Second, the measurement can be compromised by oblique reflections, as shown in Figure 3. In the case of a long acoustic path, the time-of-flight difference between the direct and off-axis reflections becomes insignificant, causing the signals to overlap and become indistinguishable.

**Figure 4:**
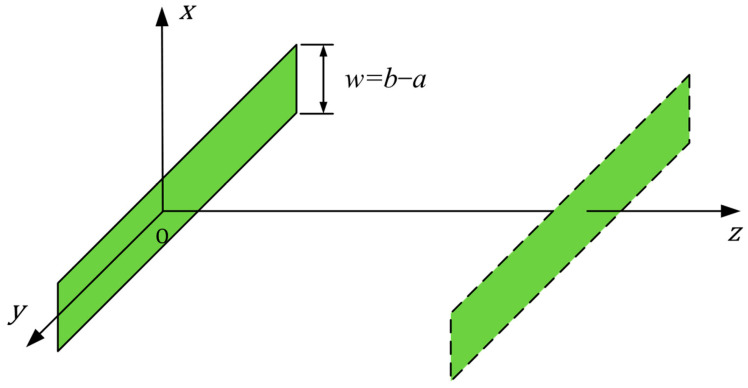
Illustration of the long-belt source assumption.

Typically, the width of a ring-shaped element is small compared to its circumference. The near-field zone is very large if the entire ring is considered the aperture. However, from a local perspective, the ring width can be considered the local aperture, which exhibits a much shorter near-field distance. Based on this concept, the long-belt source assumption is introduced to simplify the calculation. We assume that the transducer is conceptually stretched into a long rectangle (a “belt”) with a length of $\pi \left(a+b\right)$ and width of b−a, as shown in Figure 4. As the receiver is sufficiently close to the transmitter, the transducer can be considered a series of linear sources of infinite length.

The radiated sound field could be expressed as,

(4) $$p\left(x,y,z\right)=\frac{-i\omega \rho v_{0}}{2}\int_{-w/2}^{w/2}H_{0}^{\left(1\right)}\left(k\sqrt{{\left(x-x_{0}\right)}^{2}+z^{2}}\right)dx_{0}$$

where $H_{0}^{\left(1\right)}$ is zero-order Hankel function, *w = b − a* is the width of the rectangle, ω is the angular frequency, ρ is the density of the medium, $v_{0}$ is the velocity magnitude, k is the wave number, $\left(x,y,z\right)$ are the cartesian coordinate of the point of interest, and $x_{0}$ is the integral position along *x* axis.

In condition of $k\sqrt{{\left(x-x_{0}\right)}^{2}+z^{2}}\gg 1$, we can apply the far field approximation,

(5) $$H_{0}^{\left(1\right)}\left(kR\right)\approx \sqrt{\frac{2}{\pi kR}}e^{i\left(kR-\pi /4\right)},R=\sqrt{{\left(x-x_{0}\right)}^{2}+z^{2}}$$

We have,

(6) $$p\left(x,y,z\right)\approx \frac{-i\omega \rho v_{0}e^{-i\pi /4}}{2}\sqrt{\frac{2}{\pi k}}\int_{-w/2}^{w/2}\frac{e^{ikR}}{\sqrt{R}}dx_{0}$$

Since we are interested only in the area within the receiving transducer where ${\left(x-x_{0}\right)}^{2}$ is neglectable compared to $z^{2}$, we can further simplify the equation as,

(7) $$p\left(x,y,z\right)\approx \frac{\omega \rho v_{0}w}{\sqrt{2\pi kz}}e^{i\left(kz-3\pi /4\right)}$$

According to Equation (3), the response voltage could be estimated,

(8) $$V\left(\omega \right)\approx S\left(\omega \right)\frac{\omega \rho v_{0}\left(b-a\right)}{\sqrt{2\pi kz}}\pi \left(b^{2}-a^{2}\right)e^{i\left(kz-3\pi /4\right)}$$

### 2.3. Sensitivity Curve for an Annular Phased-Array Transducer

**Figure 5:**
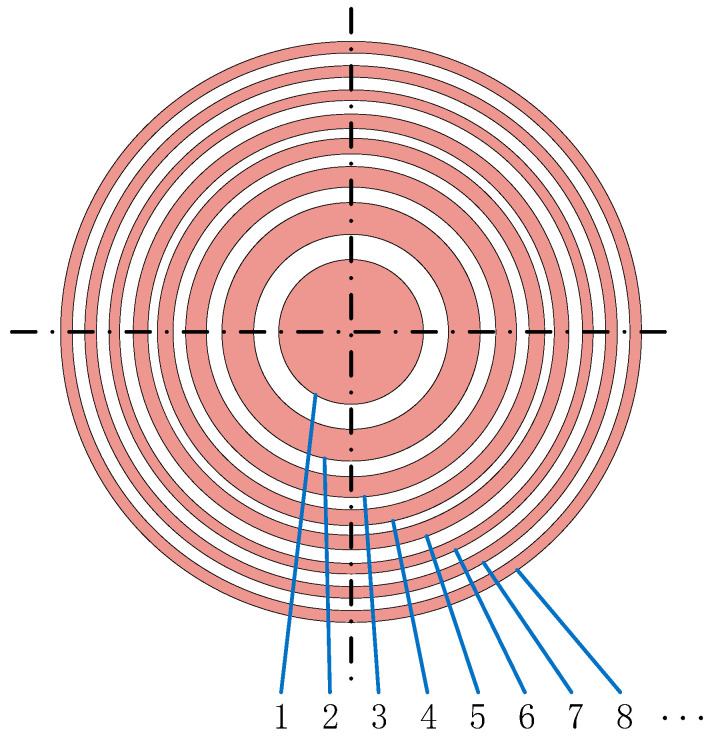
Typical elements configuration of an annular phased-array transducer.

For a linear phased-array transducer, all elements are identical, and their sensitivities are expected to be uniform. In contrast, an annular phased-array transducer is composed of multiple ring elements, each with different inner and outer radii, as shown in Figure 5. Consequently, the sensitivity is not uniform across all elements.

From Equation (8), it can be inferred that the response voltage is proportional to the product of the width and the area of a ring-shaped element.

(9) $$V_{i}\approx K\left(b_{i}-a_{i}\right)\left(b_{i}^{2}-a_{i}^{2}\right)$$

where *i* is the element number, $V_{i}$ is the amplitude, *K* is the coefficient related to the measurement condition, *b_i_* and *a_i_* are the outer and inner radii, respectively.

For impedance matching, most annular phased-array transducers are designed with equal-area rings. Therefore, the sensitivity of each element is expected to be linearly proportional to its width.

It is important to emphasize that the purpose of proposing the Long-belt source assumption model is not to replace high-fidelity numerical simulation methods like the Finite Element Method (FEM) or commercial software platforms such as CIVA. While these methods can model the acoustic field with high accuracy, they typically demand significant computational resources and time, making them impractical for routine quality control or calibration tasks that require rapid, repeated evaluation of dozens of elements. In contrast, the core advantage of the Long-belt source assumption, as an analytical model, lies in its computational efficiency. It reveals the dominant linear relationship between the response amplitude and the radial width of the ring element at a very low computational cost. As will be demonstrated by the simulation results in this paper, this approximation provides sufficient accuracy for thin ring elements, thus offering an efficient and practical evaluation tool for industrial applications.

## 3. Materials and Methods

### 3.1. Numerical Calculation

Before the experiments, numerical calculations were performed using the Rayleigh integral and the long-belt source assumption model.

For validation, the parameters of a realistic transducer were used in the calculations. The transducer was custom-manufactured by IMASONIC (Voray-sur-l’Ognon, France) and was composed of 32 elements: a central circular disk and 31 surrounding rings. The total diameter is 35 mm, and the gap between adjacent elements is 0.1 mm. The nominal frequency is 10 MHz for each element, and the bandwidth is 60%. The dimensions of each element are provided in the Supplementary Materials.

**Figure 6:**
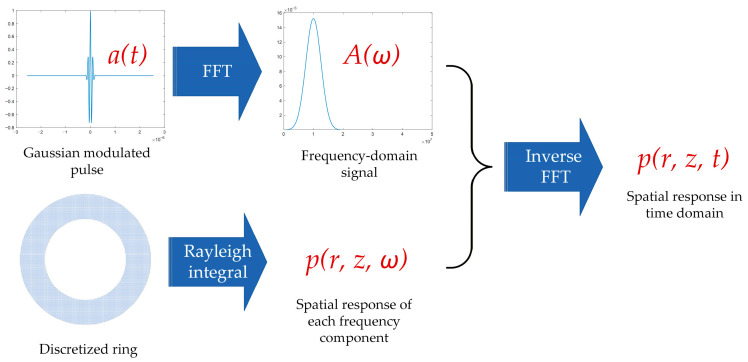
Schematic diagram of the numerical calculation workflow.

The transmission and reception of ultrasound are fundamentally achieved through the interaction between the transducer and the sound field, which is closely related to the transducer’s shape and size. Therefore, analyzing the sound field distribution of each element provides an overall insight into the sensitivity variation. The transverse sound field distributions of the elements were calculated through the Rayleigh integral as expressed in Equation (2). Since the pitch–catch mode was used in the simulation, the total two-way travel path was taken into account to ensure consistency with the pulse–echo mode in which the subsequent experiments were conducted. Therefore, the distance between the transducer and the computational plane was set to 40 mm, which is twice the distance of the water path used in the experiments. To calculate the integral numerically, each element was discretized into a series of 0.02 mm × 0.02 mm units. To account for the frequency domain contribution, a Gaussian-modulated pulse was utilized as the excitation source, with a central frequency of 10 MHz and a bandwidth of 60%. A fast Fourier transform was then performed on the pulse. The sound field of each frequency component was calculated numerically using the Rayleigh integral, and the synthesized results were obtained via an inverse FFT. A schematic diagram of the numerical calculation workflow is shown in Figure 6.

**Figure 7:**
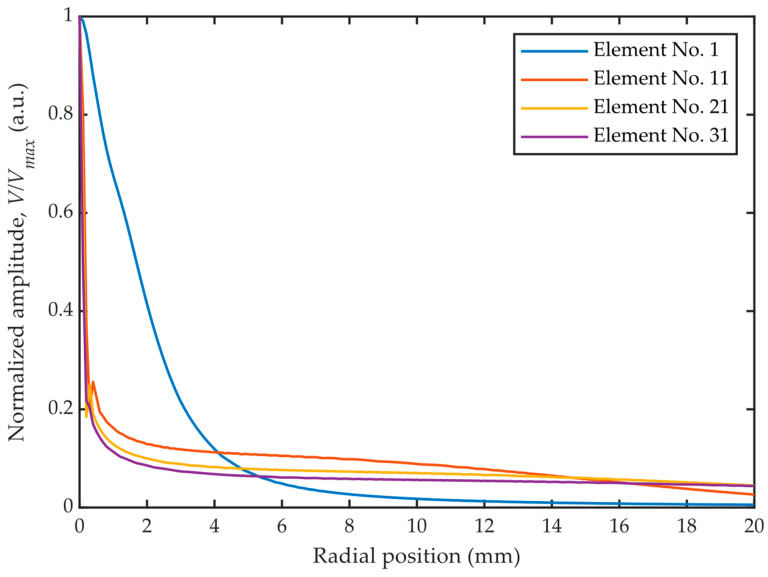
Normalized amplitude versus the radial position of element #1, #11, #21, and #31.

The normalized amplitude versus radial position curves for elements #1, #11, #21, and #31 are shown in Figure 7. As the element diameter increases, the sound field becomes more concentrated in the near-axis region and flatter in the off-axis region. Moreover, the sound field distribution changes significantly from element to element due to variations in their shapes and sizes. Notably, element #1, the only circular element of the transducer, exhibits the most diverged sound field due to its poor directivity compared to the other ring-shaped elements.

In order to investigate the influence of the geometry, the 2D radiated acoustic field distribution of a ring-shaped transducer is simulated with different inner and outer radius ratios. The outer radius of the transducer is fixed at 2.715 mm, which is the same as element 1#. By changing the inner radius between typical values of 0, 1/4, 1/2, and 3/4, different radial widths are simulated. As a special case, the transducer turns into a circular one when the inner and outer radii ratio is 0.

**Figure 8:**
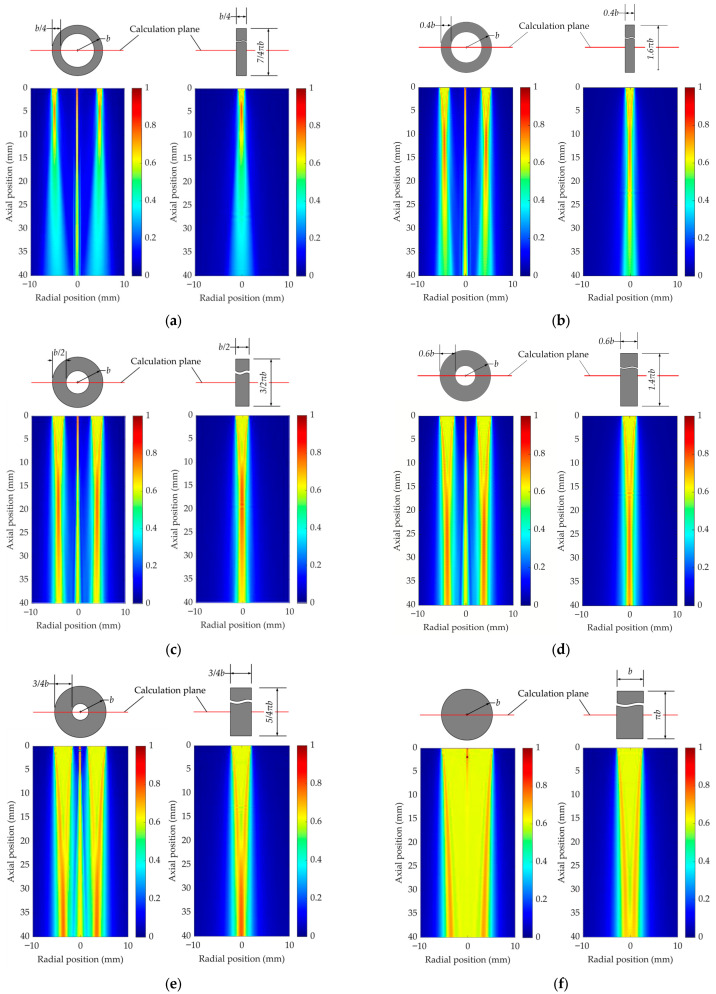
2D acoustic field distribution comparisons between the ring-shaped transducers with the ring width to the outer radius ratios of (**a**) 0.25, (**b**) 0.4, (**c**) 0.5, (**d**) 0.6, (**e**) 0.75, (**f**) 1, and the equivalent rectangular transducers.

For comparison, the acoustic field distributions of the equivalent rectangular elements are also simulated, which are derived by unwrapping and stretching the corresponding ring-shaped elements as shown in Figure 4. The results of the 2D sound field distributions are shown in Figure 8, which were calculated using a custom script developed in the Octave GNU environment.

It is important to clarify that the 2D sound field distributions for the transducers shown in Figure 8 were not calculated using Equation (7), as that equation represents the integrated response voltage. Instead, these 2D field plots were simulated by numerically solving the 2D Rayleigh integral for a ring-shaped or rectangular source of equivalent width and length, providing a direct visual comparison of the radiated field patterns to support the long-belt source assumption.

In Figure 8a through Figure 8c, the beam pattern of the ring-shaped transducer shows symmetry, and the sound field energy on the central axis is very concentrated. The main reason is that the distance from each point on the ring to the central axis is almost the same due to the small ring width. As a result of coherent interference, the ultrasound is greatly enhanced on the central axis. Away from the central line, the sound beam radiated from where the annular transducer is located is similar to that of a piston source. Due to the small width, the acoustic vibration and propagation of a component unit are not affected by other parts on the ring, which is very similar to that of a long-belt source. It can be obviously seen that when the left or right half of the image is focused, the sound field distribution is almost identical to that of an equivalent rectangular transducer. After a longer distance of propagation, the sound beam will spread and intersect at the central axis. The sound paths from different parts of the ring tend to be close, and the amplitude near the axis will be intensified as a result of interference. This explains why the beam pattern of the ring-shaped transducer tends to converge from the sides to the center at an axial position greater than 30 mm.

In Figure 8d through Figure 8f, the sound field distribution grows more similar to that of a circular piston transducer. As the ring width increases, the energy of the sound field is dispersed in a large radial range rather than converged on the central axis. If the ring width is regarded as the aperture to calculate the near-field length, the beams from different parts of the ring overlap and interact before the near-field point is reached.

The sound field distribution simulations suggest that the long-belt assumption applies to ring-shaped transducers with an inner to outer radii ratio greater than 0.5. And the thinner the ring is, the more accuracy we can get with the model.

**Figure 9:**
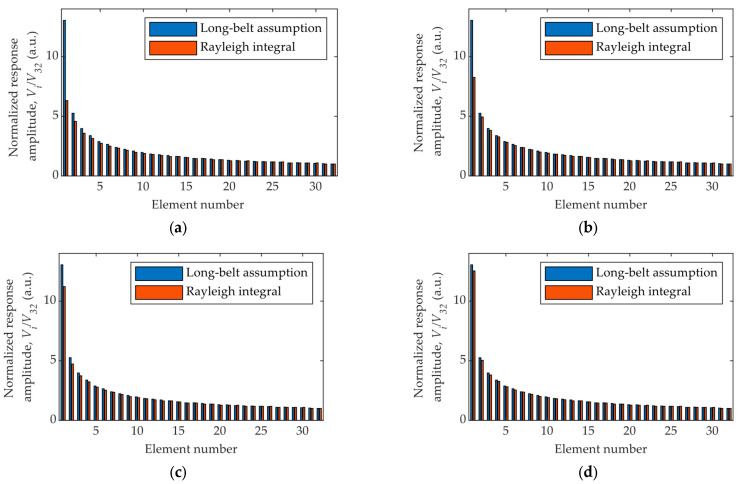
Normalized response amplitude of each element at the transmitting-receiving distance of (**a**) 40 mm; (**b**) 60 mm; (**c**) 80 mm; (**d**) 100 mm.

Since the radial variation of the sound field is neglected in the Long-Belt assumption model, the amplitude along the radial direction for each element is constant and therefore meaningless. This is the reason the Long-Belt assumption model is not used in the sound field calculation. Even so, the responses of the elements can be obtained by the model, which shows the sensitivity variation. The response amplitude of each element is calculated using both the Long-Belt assumption model and the Rayleigh integral, as shown in Figure 9. Given that we are focused on the element-to-element difference rather than the absolute response and that the long-belt assumption model is more accurate for rings with larger diameters and smaller widths, a normalization is made by dividing all the response amplitudes by that of the ring element at the outermost position. To investigate the influence of the water path distance, the calculations are made for different transmitting-receiving distances.

For most elements, the long-belt assumption and the Rayleigh integral give almost the same results except for element no.1, which has the largest difference of around 60% between the two models. This is not surprising given that it is a circular element and the radiated sound field is more diverged than other ring-shaped ones. In this case, the long-belt assumption may not be perfectly applicable to the central element. Even so, as the transmitting-receiving distance increases from 40 mm to 100 mm, the difference drops from 60% to around 5%.

**Figure 10:**
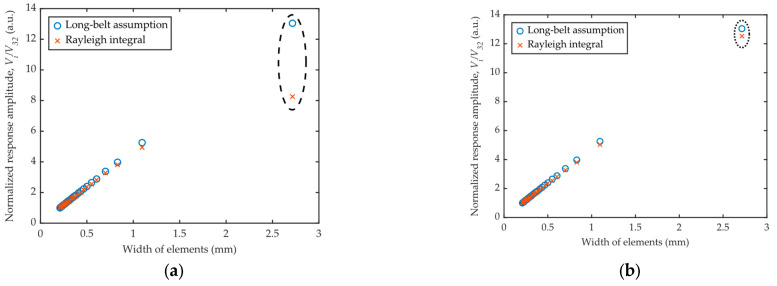
Normalized response amplitude versus the width of each element at the transmitting-receiving distance of (**a**) 40 mm and (**b**) 100 mm.

According to Equation (9), the response amplitude of a ring-shaped element is expected to be linear with its width. The curve of the normalized amplitude versus the width of each element is drawn in Figure 10. The results show obvious linearity between the amplitude and the width for all the ring-shaped elements, in both the long-belt assumption and the Rayleigh integral model.

Though time-consuming, the numerical Rayleigh integral is believed to be accurate in both near-field and far-field calculations and could be regarded as reflecting the real situation. It can be inferred that the proposed long-belt assumption model could be used to predict, with satisfactory accuracy and efficiency, the responses of ring-shaped elements within an annular phased array transducer.

### 3.2. Experimental Setup

To validate the theoretical analysis, the sensitivity of an annular phased array transducer was measured. All parameters were the same as described in Section 3.1.

**Figure 11:**
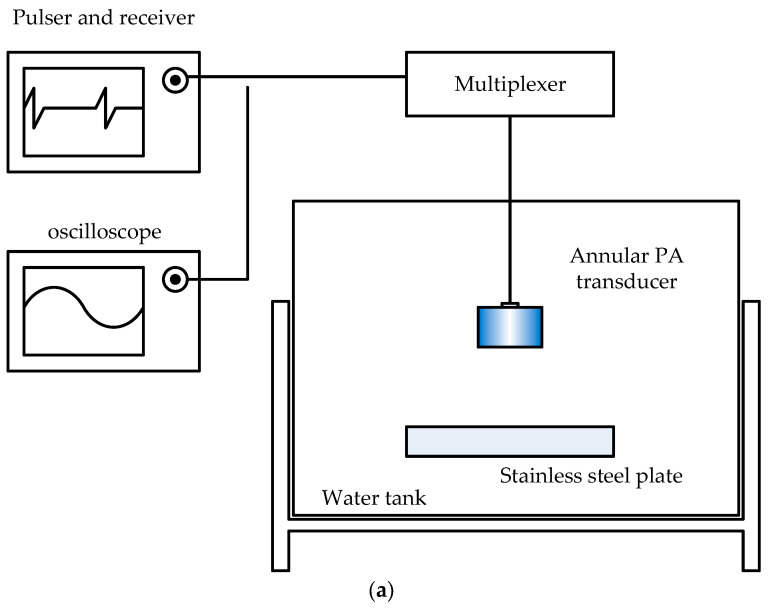

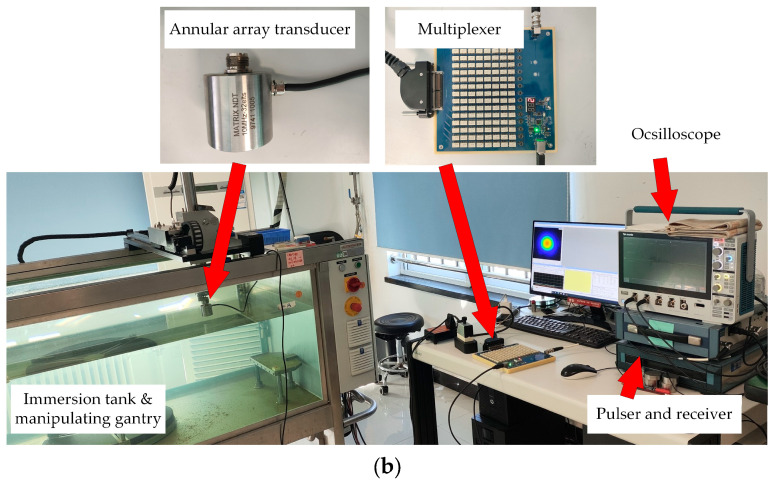
Experiment layout for measuring the sensitivity of an annular PA transducer (**a**) schematic diagram (**b**) photograph.

The standard procedure described in ISO 18563-2 for measuring and evaluating the relative pulse–echo sensitivity variation of a phased array transducer was followed. The measurement was performed using an immersion setup, as shown in Figure 11. A Model 5800 pulser-receiver (Houston, TX, USA) and a Tektronix MDO34 oscilloscope (Beaverton. OR, USA) were used to generate and capture the signals, respectively. Both instruments were connected to a multiplexer, which served as an electronic switch to select the element of interest. The multiplexer was, in turn, connected to the transducer under test, which was immersed in a water tank. A 300 mm × 300 mm stainless steel plate was placed directly beneath the transducer at a water path distance of approximately 20 mm. The water temperature was 23 °C during the experiment.

To ensure normal incidence, the transducer was aligned such that its sound beam axis was perpendicular to the planar reflector. The central element of the annular array was activated in the pulse–echo mode. The distance between the transducer and the planar reflector was adjusted to more than the near-field distance of the central element. The tilt angles of the transducer in two orthogonal planes were adjusted to maximize reflected signal from the upper surface of the planar reflector.

A negative spike pulse of approximately 70 V was used as the excitation source. By switching the multiplexer to the corresponding channel, each element was selected and excited sequentially. To ensure consistency across elements, the positions of the transducer and the reflector were kept fixed throughout the measurements. The same acquisition parameters were used for all elements. The reflected echo from the planar reflector was displayed on the oscilloscope, and the peak-to-peak voltage amplitude was measured for each element.

To assess the stability and repeatability of the measurements, the peak-to-peak voltage amplitude for each element was measured 5 independent times. Between each set of measurements, the transducer was slightly moved and then realigned. The standard deviation of the measured voltage amplitudes for all elements was calculated to be less than 2% of their mean value. This small variation indicates that the experimental setup and measurement procedure were highly stable, justifying the use of a single set of measurements to reliably determine the relative sensitivity of the elements.

## 4. Results and Discussions

**Figure 12:**
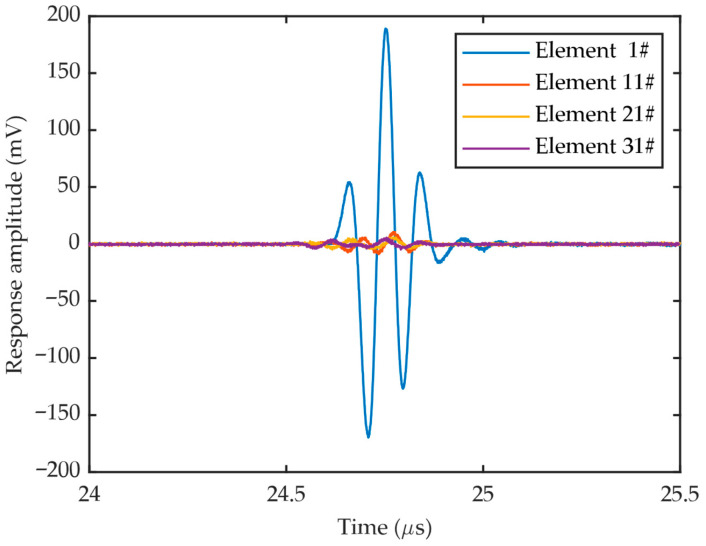
Waveforms of 4 representative elements (#1, #11, #21 and #31).

The time domain signal of each element was recorded and the response echo from the planar reflector was extracted. The waveforms of 4 representative elements are shown in Figure 12. It can be inferred that while the amplitude varies drastically, the pulse shape is almost identical across all elements.

According to ISO 18563-2, the relative sensitivity of each element is evaluated by comparing its measured raw voltage amplitude to the arithmetic mean of all elements.

(10) $$S_{el}=20lg\left(\frac{V_{el}}{V_{av}}\right)$$

where $S_{el}$ is the relative sensitivity in dB for each element, $V_{el}$ is the measured peak-to-peak voltage amplitude, $V_{av}$ is the average of all $V_{el}$.

**Figure 13:**
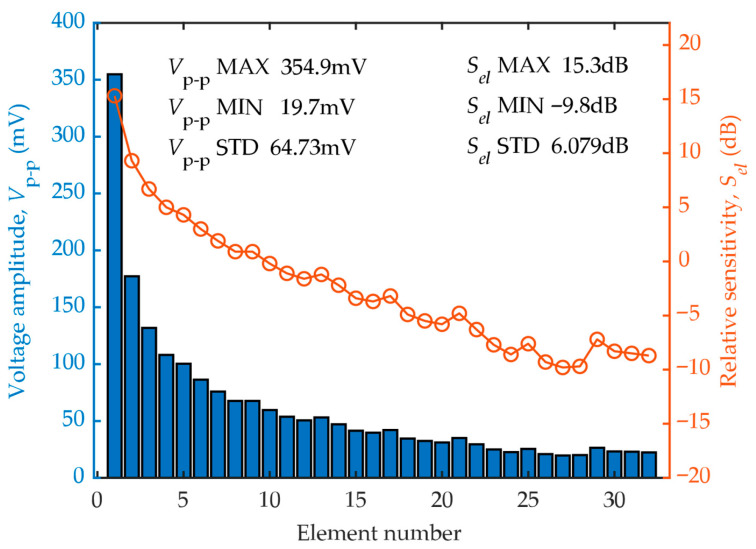
Measured peak-to-peak voltage amplitude (**left axis**) and the relative sensitivity (**right axis**) of each element.

The measured voltage amplitude and the calculated relative sensitivity for each element are shown in Figure 13.

Unlike in linear phased array transducers, the raw voltage amplitudes are not uniform across the elements of an annular array. The maximum value was nearly 18 times the minimum value, and the relative sensitivities varied from −10 dB to 15 dB. This variation is far above the 4 dB acceptance level specified in ISO 18563-2.

The preceding simulations suggest that the ratio between the maximum and minimum amplitudes could reach 13 (Figure 8). In other words, even a perfectly manufactured annular transducer with ideal dimensions and uniform electro-acoustic conversion efficiency would exhibit an element-specific sensitivity variation of 22.2 dB. Therefore, it can be inferred that the measured sensitivity variation originated primarily from the sound field differences caused by the varying shapes and dimensions of each element.

Consequently, the traditional sensitivity calculation algorithm, which is based on raw response voltage, cannot accurately reflect the intrinsic performance consistency of each element. This necessitates a new algorithm specifically for annular phased array transducers.

Based on the linear relationship between the response voltage and radial width, a new evaluation algorithm for ring-shaped elements is introduced to characterize their sensitivity independently of their geometry. The relative sensitivity for ring-shaped elements $S_{R,i}$ is defined as,

(11) $$V_{w,i}=\frac{V_{i}}{b_{i}-a_{i}}$$

(12) $$S_{R,i}=20lg\left(\frac{V_{w,i}}{V_{w,av}}\right)$$

where $V_{w,i}$ is the response voltage per unit radial width of the *i*th element, *i* is the element number, *V* is the response amplitude in voltage, *b* and *a* are the outer and inner radii, respectively, $S_{R,i}$ is the relative sensitivity for ring-shaped elements, $V_{w,av}$ is the average of all $V_{w,i}$.

**Figure 14:**
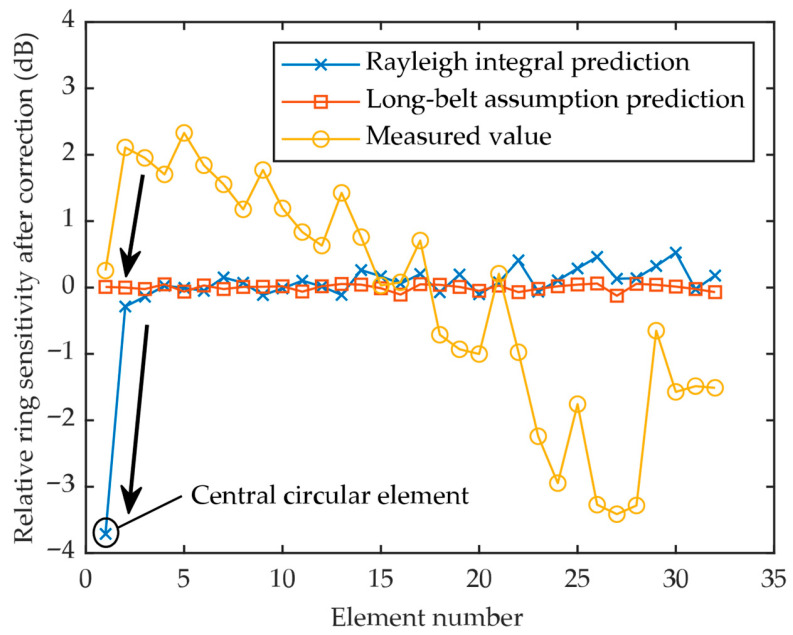
Relative ring sensitivity of all elements (Note the significant deviation for the central circular element, indicating the inapplicability of the correction algorithm to this element).

The relative ring sensitivities of all elements are shown in Figure 14. Ideally, the predicted sensitivity values for all ring-shaped elements fall within ±1 dB, as shown by the two simulation curves. In comparison, the measured values ranged from approximately −3.4 dB to 2.3 dB. The standard deviation was 1.73 dB, a significant improvement compared to the 6.079 dB from the uncorrected evaluation. According to ISO 18563-2, the relative pulse–echo sensitivity variation shall be within ±4 dB for “daisy” and “encircling” phased array transducers. These results suggest that the annular phased array transducer meets the acceptance criteria when the corrected evaluation algorithm is applied. By eliminating the sensitivity variation that originates from geometric differences, the fundamental performance consistency of the elements can be extracted and evaluated.

For the central element, the Long-belt assumption model is not applicable, as the predicted difference between the two models reaches nearly 4 dB. A significant deviation from the trend is observed for the central element in both the measured data and the Rayleigh integral prediction, whereas the Long-belt assumption model shows no such deviation. It can be inferred that the corrected sensitivity evaluation algorithm introduces significant errors for the circular element and that its application should be restricted to the ring-shaped elements.

**Figure 15:**
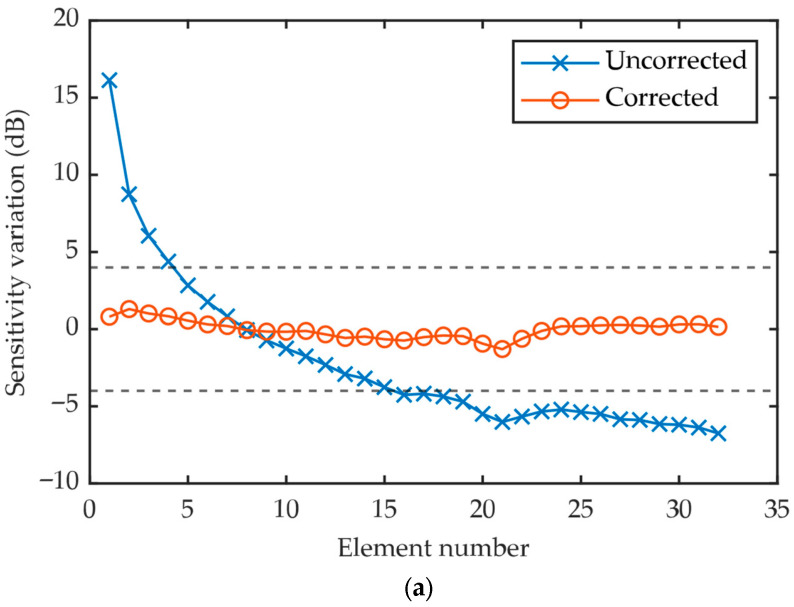

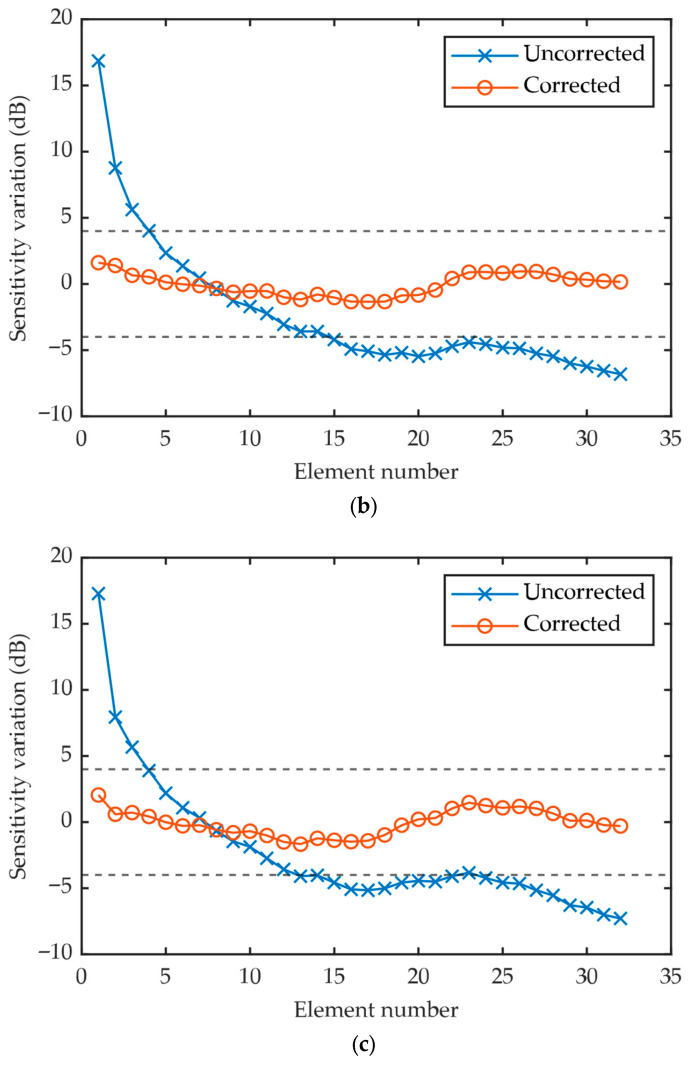
Comparison of corrected and uncorrected sensitivity variation measured at water path of (**a**) 40 mm; (**b**) 60 mm; (**c**) 80 mm.

To validate the proposed method, similar measurements were conducted with water path distances of 40 mm, 60 mm and 80 mm, as shown in Figure 15. It can be observed that the sensitivity variation evaluated through the correction algorithm drops drastically compared to the uncorrected data, regardless of the water path distance. All the corrected results fall within the ±4 dB acceptance criteria, which is a strong implication that the sensitivity consistency evaluation is depth-independent. This is a major practical advantage, as it means factory technicians do not need to perform measurements at multiple depths to certify a probe.

## 5. Benefits and Limitations

The sensitivity correction algorithm proposed in this study has significant industrial values, particularly in probe qualification and standardization. In safety-critical industries such as aerospace and energy, NDT reliability is vital, and the accurate evaluation of probe performance is fundamental to ensuring it. The potential benefits of applying the correction algorithm includes

- Solving the compliance challenge. The ISO 18563-2 standard specifies a relative pulse–echo sensitivity variation within ±4 dB for “daisy” and “encircling” probes, which includes annular arrays. However, using raw measurements directly would result in an apparent exceedance of the ±4 dB acceptance limit. By applying the proposed geometric correction, the true sensitivity variation for all ring elements can be verified to comply with the ±4 dB criterion. This demonstrates that the proposed algorithm serves as a necessary methodological bridge, enabling the proper evaluation of annular array probes against existing standards.
- Improving quality control. Manufacturers can use this algorithm to distinguish true manufacturing defects (e.g., inconsistent piezoelectric material properties, poor acoustic matching layer bonding) from expected geometric variations, leading to more accurate quality control. Evaluating probes based on raw data could lead to functional probes being incorrectly rejected for failing to meet sensitivity consistency criteria. The corrected algorithm prevents such “false negatives” caused by geometric effects, thereby saving costs for manufacturers.
- Providing accurate data for compensation. The proposed correction algorithm provides a physically sound framework for interpreting the large variation across elements in annular arrays. It decomposes the total variation into two components: a predictable, systematic variation caused by the differing element geometries, and the remaining random variation that reflects the true consistency of the elements. Based on these values, accurate correction factors for all elements can be calculated, enabling effective software compensation to balance sensitivity fluctuations. This directly enhances the NDT reliability in high-demand industries such as aerospace and nuclear power.
- Supplementing existing standards. The algorithm presented in this study could serve as a necessary procedural addendum when applying standards like ISO 18563-2 or ASTM E2904 to annular array transducers. Given its simplicity and effectiveness, the method has the potential to be formally integrated into future revisions of these standards, rather than remaining a research-level proposal.

**Figure 16:**
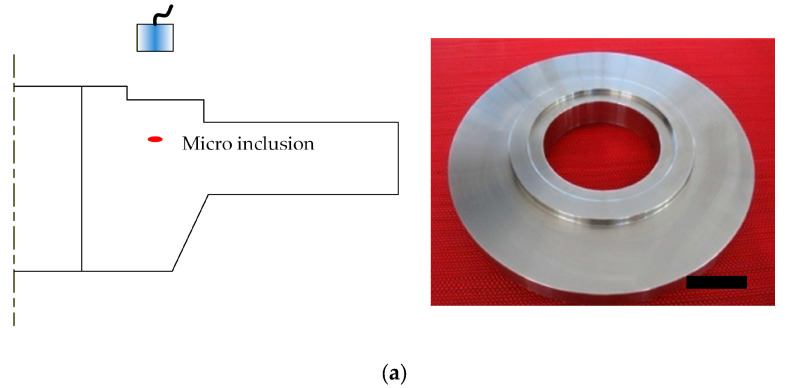

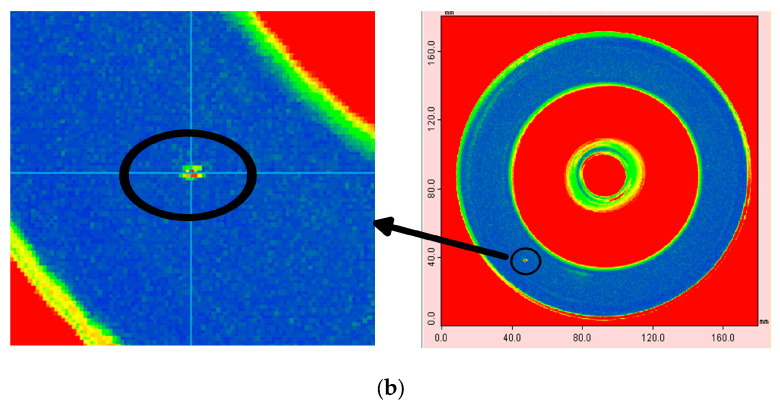
Typical C-Scan image using the annular phased array transducer with acceptable sensitivity consistency (**a**) defective disk and inspection setup; (**b**) C-Scan image with the defect indication.

As a demonstration of the efficacy of the proposed approach, a disk forging was inspected by the immersion phased array ultrasonic testing technique using a calibrated annular phased array transducer. The C-Scan image of each surface was obtained by scanning in the normal-incident longitudinal wave mode. A defect indication, which was proven to be a micro inclusion as small as 0.2 mm later, was detected and shown in the C-scan image with satisfactory SNR and resolution, as shown in Figure 16.

Despite its benefits, the proposed correction algorithm has limitations, most notably its inapplicability to the central circular element, as discussed in the preceding sections. The assumption of a “long, thin belt” is physically invalid for a solid circular disk. As a result, the correction algorithm failed to characterize the central element, which was proven by both the simulation and experiments. A potential approach to overcome this is to evaluate it as an independent circular piston transducer, or its sensitivity could be compared against a calibrated reference piston transducer of the same dimensions. In practice, a more rigorous and feasible approach is to exclude the central element from the consistency evaluation of the ring elements and perform a separate performance calibration for it.

Beyond the central element, another limitation of the model is that its accuracy decreases for rings with extreme radial widths. For example, a wide ring (with inner-to-outer radius ratio less than 0.5) will behave more like a circular element, where the curvature effect becomes significant. In some extreme cases where the radial width of the element is too small (comparable to the lateral size of PZT pillars), the model error would increase due to transverse mode coupling.

Additionally, at very short water paths, complex near-field effects may not fully align with the model’s assumptions, potentially introducing errors. However, since the near-field distance, calculated using the ring width rather than the radius as the aperture, is very short for any ring-shaped element, this should not be a problem in most cases.

It should be noted that the experimental validation in this study was performed on a specific 10 MHz transducer. Although theoretical analysis suggests that the principle of the proposed correction algorithm is frequency-independent, further experimental validation across a broader frequency range (e.g., 2.25 MHz to 15 MHz) and on different types of annular array probes is needed to fully establish the method’s universality.

## 6. Conclusions

This work investigated the element sensitivity consistency of annular phased array transducers. Through theoretical analysis, the long-belt source assumption model was proposed to describe the pitch–catch response of ring-shaped elements within an annular array. Comparative simulations were made using both the proposed model and the Rayleigh integral. The results suggest that the two models yield nearly identical normalized response amplitudes for all ring-shaped elements. However, the central circular element shows a significant discrepancy between the two models due to its unique shape and more divergent sound field. The responses of ring-shaped elements can be simulated with satisfactory accuracy and efficiency using the proposed Long-belt assumption model.

Based on these findings, a correction algorithm was proposed to evaluate the sensitivity consistency of annular phased array transducers. This algorithm uses the response voltage per unit width, rather than the total response voltage, to characterize sensitivity variation. This approach eliminates the contribution from the geometric differences of individual elements.

Experimental verification was conducted using a 32-element annular array transducer. The response voltage of each element was measured and evaluated using both the original and the corrected algorithms. The element-to-element variation decreased from a range of −10 dB to 15 dB down to −3.4 dB to 2.3 dB. The results suggest that all ring-shaped elements can be accurately evaluated with this algorithm. However, the algorithm does not apply to the central circular element due to the significant error it introduces.

The algorithm proposed in this study enables a true evaluation of sensitivity consistency for annular array probes, without requiring complicated simulations or experimental designs. The framework and logic of this method are highly compatible with existing standards such as ISO 18563-2 and ASTM E2904. Through a simple correction of the raw measurements, the sensitivity variation of an annular array can be evaluated against acceptance criteria (e.g., ±4 dB) in the same manner as for linear array probes. This approach bridges the gap between existing standards and the unique characteristics of annular array probes.

## Data Availability

The raw data supporting the conclusions of this article will be made available by the authors on request.

## Supplementary Materials

The following supporting information can be downloaded at: https://www.mdpi.com/article/10.3390/s26010025/s1, Table S1. The dimensions of each element of the transducer used in the experiments; Table S2. Measured Vpp.
