# Supplementary material for: Practical Method for Evaluating the Element Sensitivity Variation of an Ultrasonic Annular Phased Array Transducer
Source: Sensors (Basel). 2025 Dec 19;26(1):25. doi: 10.3390/s26010025 (PMC12787951; doi:10.3390/s26010025)
Supplement: Supplementary file 1 [file sensors-26-00025-s001.zip › Table S1. The dimensions of each element of the transducer used in the experiments.pdf]

Table S1. The dimensions of each element of the transducer

Element No.	Interior radius (mm)	Exterior radius (mm)	Ring width (mm)	Element area (mm^2)
1	0	2.715	--	23.16
2	2.815	3.91	1.095	23.13
3	4.01	4.84	0.83	23.08
4	4.94	5.64	0.7	23.27
5	5.74	6.345	0.605	22.97
6	6.445	6.995	0.55	23.22
7	7.095	7.595	0.5	23.07
8	7.695	8.16	0.465	23.16
9	8.26	8.695	0.435	23.17
10	8.795	9.205	0.41	23.18
11	9.305	9.69	0.385	22.97
12	9.79	10.16	0.37	23.19
13	10.26	10.615	0.355	23.28
14	10.715	11.055	0.34	23.25
15	11.155	11.48	0.325	23.11
16	11.58	11.89	0.31	22.86
17	11.99	12.295	0.305	23.27
18	12.395	12.69	0.295	23.25
19	12.79	13.075	0.285	23.16
20	13.175	13.45	0.275	23.00
21	13.55	13.82	0.27	23.22
22	13.92	14.18	0.26	22.95
23	14.28	14.535	0.255	23.08
24	14.635	14.885	0.25	23.18
25	14.985	15.23	0.245	23.26
26	15.33	15.57	0.24	23.30
27	15.67	15.9	0.23	22.81
28	16	16.23	0.23	23.29
29	16.33	16.555	0.225	23.25
30	16.655	16.875	0.22	23.17
31	16.975	17.19	0.215	23.08
32	17.29	17.5	0.21	22.95
